# Introgressions lead to reference bias in wheat RNA-seq analysis

**DOI:** 10.1186/s12915-024-01853-w

**Published:** 2024-03-07

**Authors:** Benedict Coombes, Thomas Lux, Eduard Akhunov, Anthony Hall

**Affiliations:** 1 Earlham Institute, Norwich, Norfolk, NR4 7UZ UK; 2 Plant Genome and Systems Biology, Helmholtz Zentrum München, Neuherberg, Germany; 3 Department of Plant Pathology, Kansas State University, Manhattan, KS USA

**Keywords:** Wheat, RNA-seq, Reference bias, Genomics, Introgressions, Polyploidy

## Abstract

**Background:**

RNA-seq is a fundamental technique in genomics, yet reference bias, where transcripts derived from non-reference alleles are quantified less accurately, can undermine the accuracy of RNA-seq quantification and thus the conclusions made downstream. Reference bias in RNA-seq analysis has yet to be explored in complex polyploid genomes despite evidence that they are often a complex mosaic of wild relative introgressions, which introduce blocks of highly divergent genes.

**Results:**

Here we use hexaploid wheat as a model complex polyploid, using both simulated and experimental data to show that RNA-seq alignment in wheat suffers from widespread reference bias which is largely driven by divergent introgressed genes. This leads to underestimation of gene expression and incorrect assessment of homoeologue expression balance. By incorporating gene models from ten wheat genome assemblies into a pantranscriptome reference, we present a novel method to reduce reference bias, which can be readily scaled to capture more variation as new genome and transcriptome data becomes available.

**Conclusions:**

This study shows that the presence of introgressions can lead to reference bias in wheat RNA-seq analysis. Caution should be exercised by researchers using non-sample reference genomes for RNA-seq alignment and novel methods, such as the one presented here, should be considered.

**Supplementary Information:**

The online version contains supplementary material available at 10.1186/s12915-024-01853-w.

## Background

Quantification of gene expression using RNA-seq is a fundamental technique in genomics research. It has been employed in numerous publications across a range of biological systems to identify candidate genes underlying traits of interest, uncover transcriptional pathways and networks, and investigate hypotheses relating to gene and transcriptional evolution and adaptation. In RNA-seq experiments, mRNA, which represents a snapshot of the expression of each gene at the time of sampling, is extracted from the biological sample, converted to cDNA and sequenced. The number of resulting RNA-seq reads deriving from each gene/transcript are quantified, with the number of reads proportional to the level of expression of that gene/transcript. Quantifying the expression level of each transcript and/or gene typically involves alignment of sequencing reads to the reference genome or transcriptome of the sequenced species using spliced alignment tools such as HISAT2 [1] and STAR [2] or pseudoalignment tools such as kallisto [3] and Salmon [4]. Despite these tools typically being developed and benchmarked with human data, they are widely used across numerous biological systems, often without consideration for how they will behave with specific challenges the genomes of different species present.

Making meaningful inferences from RNA-seq data relies upon the accuracy of alignment and quantification; downstream analyses and subsequent interpretation assumes that the estimated gene expression reflects actual gene expression in the biological samples. However, nucleotide variation in the coding region of genes between the sequenced sample and the reference genome/transcriptome leads to errors in read assignment during the alignment/pseudoalignment step. Some reads may be unassigned, while others may be assigned to the wrong locus. This source of error is widely known as reference bias as transcripts derived from alleles present in the reference sequence will be quantified more accurately [5].

The reduction in accuracy caused by reference bias has the potential to negatively impact downstream analyses and lead to incorrect findings. For example, Thorburn et al. [6] demonstrated how using a single reference genome to map sequencing data from genetically diverse individuals causes reference bias that negatively impacts downstream analyses in population genomic studies. While this study looked at mapping DNA reads, the same can be assumed to be true about RNA-seq data. Zhan, Griswold and Lukens [7] found that accurate estimates of transcript abundances from RNA-seq reads in maize are strongly affected by reference bias. By reanalysing RNA-seq data from a B73xMo17 recombinant inbred line population, they found that the detection of around 50% of expression quantitative trait loci (eQTLs) alleles depended on which reference genomes was used: B73 or Mo17. As the previous study [8] used B73 as the reference, Zhan et al. [7] estimated that 50% of the detected eQTLs may be false positives. Munger et al. [9] found that mapping RNA-seq reads to individualised genomes instead of a single reference genome substantially increased the accuracy of eQTL assignment in mouse from 88.2 to 98.3%, removing false positive results that appeared when using a single reference genome.

The impact of reference bias in RNA-seq analysis has not been assessed in complex polyploid genomes such as wheat despite these genomes having characteristics that may increase the extent and degree of reference bias relative to species with simpler genomes. Polyploidisation increases the number of alleles per gene, typically resulting in a pair of alleles, known as homoeologues, in each subgenome; however, subsequent gene duplications or deletions can change the relative copy number of homoeologues between the subgenomes. As RNA-seq reads are derived from all subgenomes at once, read assignment must be able to distinguish reads deriving from homoeologues. Accurate discrimination of wheat homoeologue RNA-seq reads has been demonstrated with both pseudoalignment [10, 11] (99.9% accuracy) and alignment-based (98% accuracy) [11] methods when mapping reads back to the genome from which they derived. However, when mapping reads from a different genotype, unequal divergence between homoeologues relative to the reference genome may compromise the accuracy of the expression balance estimation between homoeologues. Being able to accurately estimate homoeologue expression balance is important for wheat research as variation in the relative mRNA expression of homoeologues within a triad may confer phenotypic plasticity [10] and variation in agronomic traits, the understanding of which has important applications for crop improvement.

Introgression events, the introduction of genetic material from one species to another [12], are common among plants; in fact, its frequency is thought to be higher in plants than in animals, due to higher rates of interspecific hybridisation success [13]. Additionally, novel genetic variation is commonly introgressed into plants by breeders and researchers for crop improvement [14]. Several studies have demonstrated how common introgressions are in wheat accessions with some accessions being comprised of up to 34% introgressed material [15–19]. The production of chromosome-level genome assemblies of modern elite wheat cultivars confirmed this, revealing introgressions from wild and domesticated relatives, including species outside of the *Triticum* and *Aegilops* genera, present in one or multiple cultivars [20, 21]. These introgressions introduce greater sequence divergence between varieties than observed between varieties at non-introgressed regions; this increased divergence likely leads to an increased proportion of reads that are unable to be assigned correctly.

Using simulated and experimentally generated RNA-seq data, we identify non-trivial levels of reference bias in RNA-seq mapping in wheat which can largely be attributed to introgressions. This leads to incorrect estimates of relative expression between homoeologues and incorrectly called differences in expression between cultivars. By constructing a pantranscriptome reference composed of Chinese Spring transcripts and transcripts from the assemblies generated as part of the 10+ wheat genomes project [20], we demonstrate how reference bias caused by divergent alleles can be reduced.

## Results

### *Reference bias in wheat is driven by divergent genes introduced *via* introgressions and results in underestimation of gene expression*

To explore the impact of reference bias on the quantification of gene expression in wheat, we simulated 1000 read pairs from each high-confidence (HC) gene in Chinese Spring RefSeq v1.1 and the nine chromosome-level genome assemblies generated as part of the 10+ wheat genomes project [20, 22] if the longest transcript of the gene is at least 500 bp. These reads were pseudoaligned or aligned to the Chinese Spring reference transcriptome or genome using kallisto or STAR, respectively. These algorithms represent pseudoalignment and alignment-based methods and are among the most commonly used tools for RNA-seq quantification in the wheat community.

**Fig. 1:**
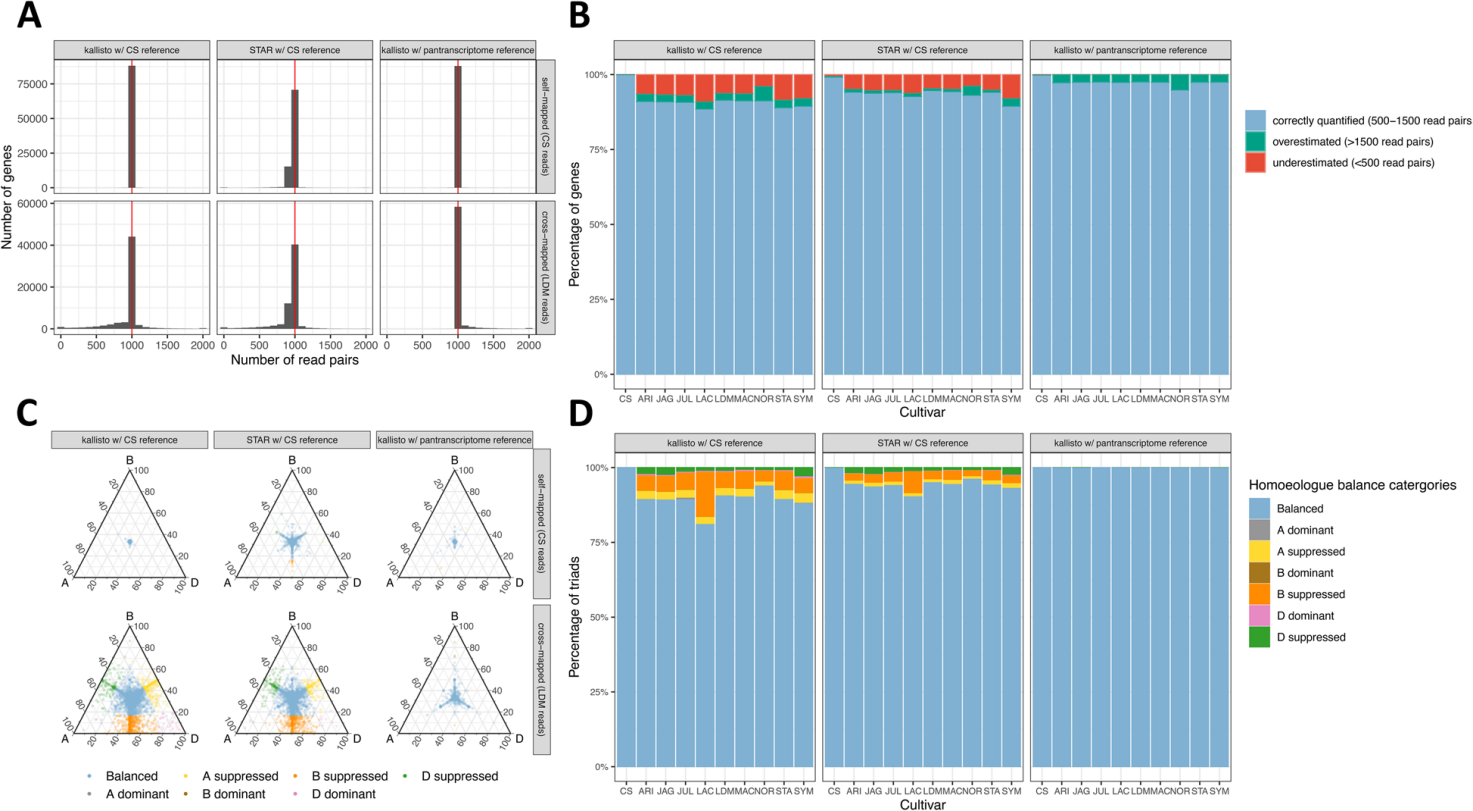
Assessing the extent of reference bias in wheat.** A** Distribution of read counts when self-mapping Chinese Spring simulated reads or cross-mapping Landmark simulated reads. Comparing STAR and kallisto using the Chinese Spring RefSeq v1.0 reference and RefSeq v1.1 transcriptome and kallisto using the pantranscriptome reference. **B** Percentage of genes with expression estimated correctly, expression underestimated (< 500 read pairs) and expression overestimated (> 1500 read pairs) for simulated reads from 10 cultivars aligned to Chinese Spring with kallisto and STAR or to the pantranscriptome reference with kallisto. **C** Balance of homoeologue expression across triads when self-mapping Chinese Spring or cross-mapping Landmark simulated reads, comparing STAR and kallisto using the Chinese Spring RefSeq v1.0 reference and RefSeq v1.1 transcriptome and kallisto using the pantranscriptome reference. Each point on the ternary plot represents one triad. Points towards a corner indicate dominant expression of that homoeologue, and points opposite a corner indicate suppression of that homoeologue. **D** Percentage of triads in each expression category, using simulated reads from 10 cultivars aligned to Chinese Spring with kallisto and STAR or to the pantranscriptome reference with kallisto

Mapping Chinese Spring reads to Chinese Spring, hereafter referred to as self-mapping, yields very accurate estimates of gene expression, with kallisto slightly outperforming STAR (Fig. 1a, b, Additional file 1: Table S1). Using kallisto, 88,401/88,443 (99.95%) of genes were correctly quantified (between 500 and 1500 read pairs). Thirty-two genes were underestimated (< 500 read pairs) and 10 genes were overestimated (> 1500 read pairs). Using STAR, 87,689/88,443 (99.15%) were correctly quantified with 504 and 250 genes underestimated and overestimated, respectively.

Mapping reads generated from the other cultivars to Chinese Spring, hereafter called cross-mapping, yielded much less accurate estimation of gene expression with a skew towards underestimation (Fig. 1a, b, Additional file 1: Table S1). The percentage of genes correctly quantified ranged from 55,773/63,001 (88.53%) for Lancer, with 5700 (9.05%) and 1528 (2.43%) under- and overestimated, respectively, to 58,468/64,077 (91.2%) for Norin61, with 2527 (3.94%) and 3082 (4.81%) genes under and overestimated, respectively. For cross-mapping, unlike self-mapping, STAR appears to perform better than kallisto; the proportion of correctly quantified genes ranged from 58,390/63,001 (92.68%) for Lancer, with 3916 and 695 under and overestimated, respectively, to 59,648/64,077 (93.1%) for Norin61, with 2450 (3.82%) and 1979 (3.09%) genes under and overestimated, respectively.

To explore the effect of reference bias on the quantification of homoeologue expression balance, we calculated the proportion of triads belonging to each category that defines a different state of relative homoeologue expression. As reads were simulated evenly across genes, all triads should be classified as balanced; therefore, triads classified as imbalanced (one or two homoeologues with expression greater than the other(s)) are considered incorrectly classified. The percentage of correctly classified triads varies between 80.97% (Lancer) and 93.84% (Norin61) using kallisto and between 90.23% (Lancer) and 96.12% (Norin61) using STAR (Fig. 1c, d, Additional file 1: Table S2). Across the cultivars, triads incorrectly classified as suppressed, where one homoeologue is estimated to be expressed less than the others, were far more common than triads incorrectly classified as dominant, where one homoeologue is estimated to be expressed more highly than the others (Fig. 1d, Additional file 1: Table S2). This reflects how the reference bias leads to more underestimated than overestimated genes.

The B subgenome has the most, and the D subgenome the fewest, number of triads incorrectly classified as suppressed. This is in line with observations of greater diversity in the A and B subgenomes, with the B subgenome having the highest [16]. This difference is largely caused by gene flow from wild tetraploid *T. dicoccoides* to *T. aestivum* during the history of its cultivation, without comparable gene flow to the D subgenome [17, 19, 23]. This finding suggests the historic gene flow from tetraploid wheat likely contributes to reference bias in RNA-seq analyses.

**Fig. 2:**
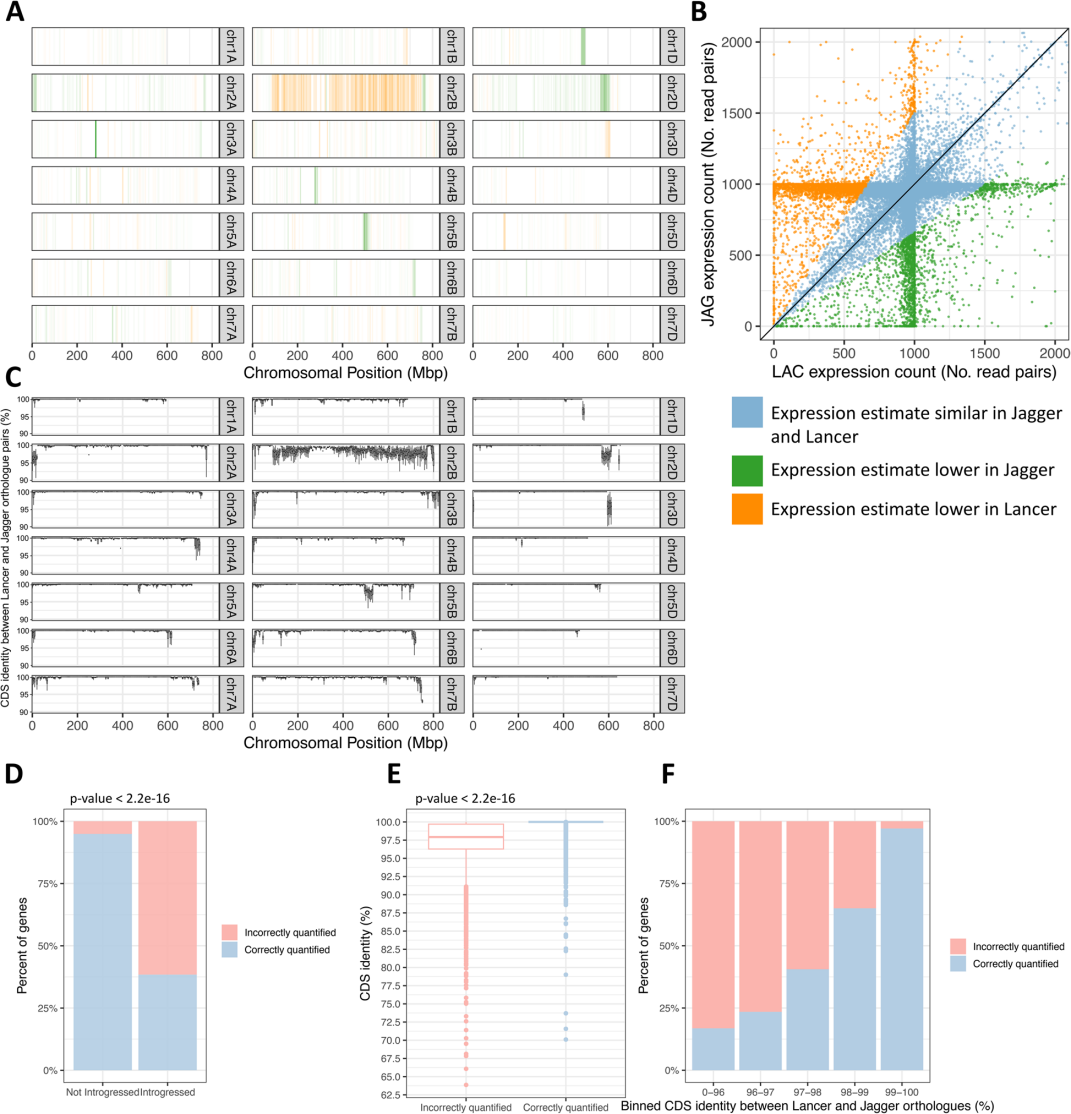
The impact of reference bias on expression differences between cultivars and enrichment of incorrectly quantified genes within introgressions. **A** The distribution of incorrectly quantified genes in 5-Mbp windows, coloured by the cultivar in which the estimated expression is lower; orange blocks are underestimated in Lancer compared to Jagger, while green blocks are underestimated in Jagger compared to Lancer. The reads are aligned using STAR as this outperformed kallisto for cross-mapping. **B** Expression counts for Lancer-Jagger orthologue pairs. Genes are considered incorrectly quantified if their estimated read count is 1.5 × or 1/1.5 × the other cultivar. **C** CDS nucleotide identity between Lancer and Jagger 1-to-1 orthologue pairs, binned into 5-Mbp genomic windows based on Chinese Spring RefSeq v1.0. **D** Percentage of genes incorrectly quantified and correctly quantified in characterised introgressed regions and regions not characterised as introgressed. **E** CDS nucleotide identity between Lancer and Jagger 1-to-1 orthologue pairs for those that are incorrectly quantified and those that are correctly quantified. **F** Percentage of genes incorrectly quantified and correctly quantified, split into bins of different levels of CDS nucleotide identity

To explore the extent of errors when comparing two cultivars mapped to a common reference, we compared the estimated expression of Lancer and Jagger genes, whose simulated reads were both aligned to Chinese Spring using STAR (Fig. 2a, b). Genes with read counts > 1.5 × or < 1/1.5 × compared to the other cultivar were classified as incorrectly quantified. Using STAR, 4791/60,338 (7.94%) genes were incorrectly quantified between the two cultivars; of these genes, 2747 and 2044 genes had a lower read count in Lancer and Jagger, respectively.

We observed a clear overlap between clusters of incorrectly quantified genes and regions of divergence between the cultivars (Fig. 2a, c), identified by blocks of reduced CDS nucleotide identity between pairs of orthologues between Lancer and Jagger. Such gene-level divergence is indicative of introgressed material; indeed, several of these blocks correspond to previously characterised introgressions. These introgressions include (coordinates based on Chinese Spring RefSeq v1.0) the following: *Aegilops ventricosa* introgression in Jagger (chr2A:1–24,643,290) [20, 21, 24]; *Triticum timopheevii* introgression in Lancer (chr2B:89,506,326–756157100) [20, 21]; *Aegilops comosa* introgression in Jagger (chr2D:570,141,481–613325841) [21]; and a *Thinopyrum ponticum* introgression in Lancer (chr3D:591,971,000–615552423) [20, 21]. 1881/3054 (61.59%) of introgressed genes (those belonging to one of the four previously characterised introgressions listed above) were incorrectly quantified between the two cultivars, compared to 2910/57,284 (5.08%) non-introgressed genes incorrectly quantified (Fig. 2d; chi-squared *p*-value < 2.2e − 16). Genes with an introgressed copy in Lancer tend to be underestimated in Lancer and genes with an introgressed copy in Jagger tend to be underestimated in Jagger.

In further support of CDS divergence being a predominant contributing factor to incorrect quantification, we found that incorrectly quantified genes have a mean CDS identity between orthologue pairs of 97.3% compared to a mean of 99.9% for genes correctly quantified (Fig. 2e; *p*-value < 2.2e − 16; 95% confidence interval ranges from 2.45 to 2.63). The percentage of genes incorrectly quantified ranges from 83.2% for genes with < 96% CDS identity between orthologues to just 2.9% for genes with ≥ 99% identity between orthologues (Fig. 2f).

### Reducing reference bias by constructing a pantranscriptome reference

**Fig. 3:**
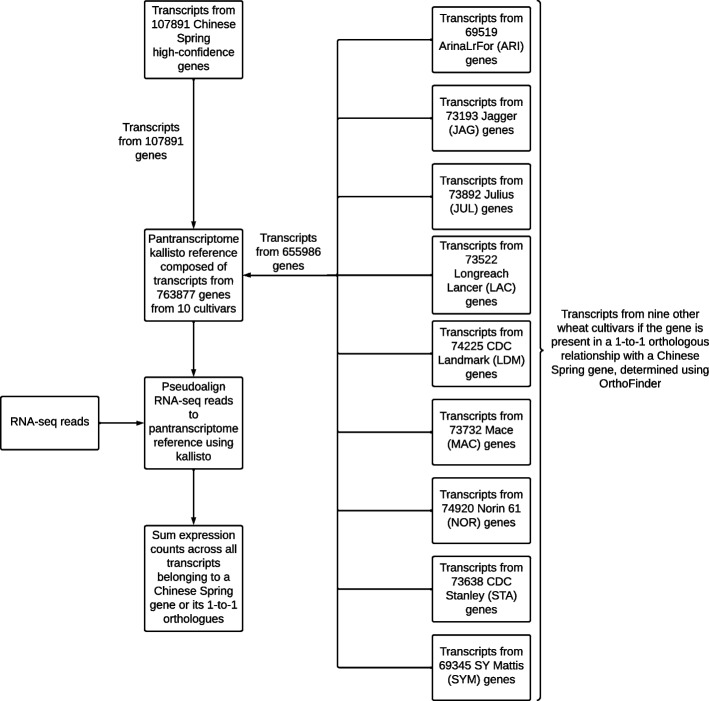
Creation of the pantranscriptome reference and how RNA-seq reads are aligned to it

The 10+ wheat genomes project generated chromosome-level de novo assembled genomes for nine wheat cultivars in addition to the reference cultivar Chinese Spring [20]. These include numerous introgressions that are the predominant source of reference bias we observe. High-quality gene annotations for these genome assemblies have been produced [22]. We constructed a pantranscriptome reference by taking the transcripts from the 107,891 Chinese Spring HC genes and adding transcripts from the nine cultivars with a chromosome-level genome assembly generated as part of the 10+ wheat genomes project [20] if that transcript’s gene exists in a 1-to-1 relationship with a gene from Chinese Spring, based on OrthoFinder [25] orthogroup assignments. This resulted in a set of transcripts from 763,877 genes from 10 cultivars, 107,891 from Chinese Spring and a mean of 72,887 from each of the nine other cultivars (Fig. 3). A total of 80,211 Chinese Spring genes had at least one 1-to-1 orthologue in another cultivar, while 59,639 Chinese Spring genes had a 1-to-1 orthologue in all nine other cultivars (Additional file 2: Fig. S1). The pantranscriptome reference was used as the transcriptome reference for kallisto pseudoalignment. After pseudoalignment, read counts and TPMs were summed across all transcripts corresponding to a given Chinese Spring gene. Kallisto splits read counts evenly across transcripts with an identical match so redundancy of transcripts does not cause problematic multi-mapping; all transcripts corresponding to a gene can thus be added.

To ensure using this pantranscriptome reference does not introduce any additional mapping errors from adding redundant transcripts, we compared quantified expression counts between four difference references: Chinese Spring, the pantranscriptome reference, Chinese Spring plus the Landmark transcripts from genes in a 1-to-1 relationship with a Chinese Spring gene, and the pantranscriptome reference without the Landmark transcripts. The simulated reads from Landmark were used for pseudoalignment. Of these four references, the pantranscriptome reference performed the best, with 97.53% of genes correctly quantified. Chinese Spring plus Landmark transcripts were very similar, with 97.50% of genes correctly quantified. This demonstrates that adding redundant transcripts and summing the read counts does not introduce errors in the kallisto mapping. Using the pantranscriptome reference without Landmark transcripts resulted in a slightly lower level of correct quantification, with 96.84% correctly quantified. The difference is likely due to uniquely introgressed genes in Landmark that are not present in the other cultivars. Nevertheless, due to many introgressed genes being common between cultivars, it still performed much better than just using Chinese Spring, which had 91.43% genes correctly quantified.

Using the pantranscriptome reference instead of Chinese Spring to quantify expression from the simulated RNA-seq reads resulted in much more accurate quantification for genes that were previously underestimated when cross-mapping, removing nearly all gene counts below 1000 (Fig. 1a, b). There was little change in the number of genes overquantified when cross-mapping and little difference in the distribution of read counts when self-mapping (Fig. 1a, b). The distribution of read counts shows that for Lancer, the most error-prone cultivar, the number of genes correctly quantified increased from 58,390/63,001 (92.68%) using STAR to 61,352/63,001 (97.38%) using the pantranscriptome reference. Using the pantranscriptome reference, only 2 genes remained quantified below 500 read pairs compared to 3916 genes when using the Chinese Spring reference. The number of triads correctly assigned to the balanced expression category also greatly increased when using the pantranscriptome reference (Fig. 1d). All cross-mapped cultivars had at least 99.89% triads correctly assigned as balanced; this compares to between 80.97 and 93.84% using kallisto, and between 90.23 and 96.12% using STAR to align to Chinese Spring.

Comparing Jagger and Lancer as before, this approach reduced the number of genes incorrectly quantified in one cultivar from 4971/60,338 (7.94%) to 617 (1.02%) (Additional file 2: Fig. S2). Only 23 genes (0.0381%) remain incorrectly quantified due to underestimation in one cultivar. Almost all the remaining error in both cross-mapped read counts and incorrectly quantified genes between cultivars is due to overestimation of gene expression, likely caused by copy number variation or presence/absence variation between cultivars, as opposed to divergence between orthologous gene models.

### Exploring reference bias caused by introgressions in experimentally generated RNA-seq data

Simulated RNA-seq data is unlikely to capture the complete picture of a real experiment [26]. While our simulations highlight theoretical errors, it is important to assess how reference bias impacts published findings and how using the pantranscriptome reference corrects errors in real data. We reanalysed the sequencing data generated by He et al. [11]. He et al. [11] analysed RNA-seq data from 198 diverse wheat accessions, alongside enrichment capture paired-end DNA reads, to uncover eQTLs linked with homoeologue expression bias and variation in important productivity traits. Crucially for our work, they identified a set of genes whose expression exhibited negative correlation with its homoeologue across the panel. A subset of accessions possessed lowly expressed alleles in one of the homoeologues and the presence of the lowly expressed alleles was linked to various important productivity traits. This set contains 59 genes to which we have added *ELF3-D1*. While *ELF3-D1* did not fall into the set of very negatively correlated 59 genes, it was used as case example due to its agronomic significance. Also, it still did show a negative correlation with its B homoeologue, with this expression bias associated with agronomic traits. This set of 60 genes is hereafter referred to as genes showing lack of expression correlation.

**Fig. 4:**
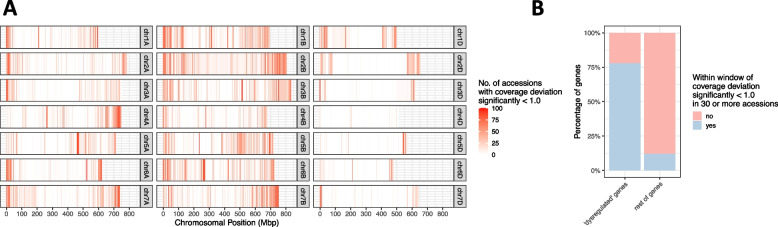
Enrichment of genes showing a lack of expression correlation in He et al. [11] within regions of divergence.** A** Chromosomal distribution of the number of accessions in each 1-Mbp genomic window which had mapping coverage deviation significantly less than 1 and are thus likely to contain divergent introgressed material or be deleted. **B** The number of genes from the set of 60 genes showing lack of expression correlation identified by He et al. [11] that are present in genomic windows identified as introgressed or deleted in 30 or more accessions

Firstly, to identify potential introgressed regions within these accessions, we mapped the enrichment capture paired-end DNA reads to Chinese Spring RefSeq v1.0 and for each 1-Mbp genomic window, calculated the mapping coverage deviation between each line and the median for that window across the accessions (Fig. 4a). Blocks of windows with coverage deviation values significantly below 1 have few reads that have mapped in this region relative to the other accessions. This is indicative of an introgression (which introduces divergent DNA that maps less well) or a deletion. We observed more divergent material in the A and B subgenomes, which is expected based on the higher levels of gene flow to the A and B subgenomes (Fig. 4a) [17, 19, 23]. The genes showing lack of expression correlation identified by He et al. [11] are enriched in genomic windows identified as introgressed or deleted (Fig. 4b), with 78.2% of these genes in a genomic window identified as introgressed or deleted in 30 or more accessions. In the rest of the genome, only 12.3% of genes are found in a genomic window identified as introgressed or deleted in 30 or more accessions.

**Fig. 5:**
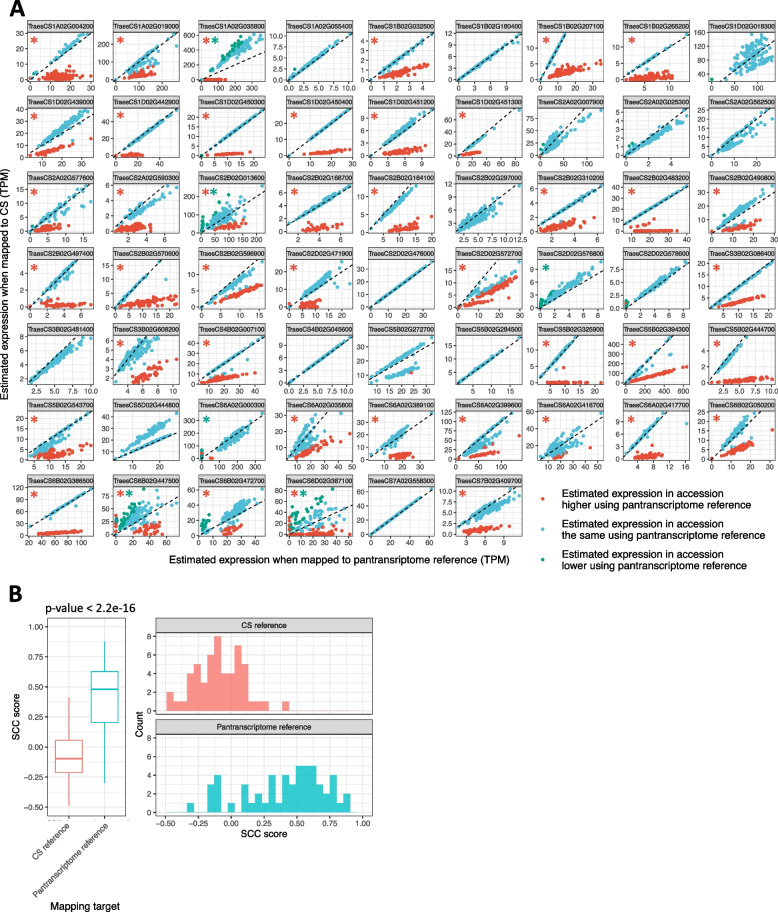
The impact of reference bias on the quantification of gene expression in the accessions sequenced by He et al. [11].** A** Estimated expression of the 60 genes identified as showing a lack of expression correlation by He et al. [11], using either the Chinese Spring RefSeq v1.1 transcriptome or the pantranscriptome reference as targets for kallisto pseudoalignment. The dashed black line represents *x* = *y*, which is the expected value if the reference is not affecting the estimation of gene expression. An accession lying on this dashed line has this gene’s expression estimated the same when using each reference. Red dots and green dots represent accessions in which a given gene has a TPM value < 50 or > 150%, respectively, when mapping to Chinese Spring than when mapping to the pantranscriptome reference. A red star indicates that in 25 or more accessions, the gene has an estimated expression less than half when mapping to Chinese Spring compared to when mapping to the pantranscriptome reference. A green star indicates that in 25 or more accessions, the gene has an estimated expression more than double when mapping to Chinese Spring compared to when mapping to the pantranscriptome reference. **B** Spearman’s correlation coefficient (SCC) between homoeologue pairs where one homoeologue is in the set of genes showing a lack of expression correlation identified by He et al. [11]. SCC scores were computed between AB, AD and BD homoeologue pairs and the lowest score was used. Triads in which any of the homoeologues were not present in the RefSeq v1.0 HC gene annotation were excluded. The significance of the difference between SCC scores when using the Chinese Spring reference compared to when using the pantranscriptome reference was calculated using a two-tailed *t*-test with no assumption of equal variance

To explore the impact of the pantranscriptome reference on estimated expression, we pseudoaligned the leaf RNA-seq data from the 198 wheat accessions to both Chinese Spring and to the pantranscriptome reference. Kallisto was used for aligning to Chinese Spring instead of STAR for consistency with the analysis by He et al. [11]. 43/60 (71.7%) of genes showing lack of expression correlation (Fig. 5a) have, in 25 or more accessions, an estimated expression less than half when mapping to Chinese Spring compared to when mapping to the pantranscriptome reference. These are likely introgressed genes whose expression is underestimated when using Chinese Spring as the reference. 6/60 (10.0%) of the genes have, in 25 or more accessions, an estimated expression more than double when mapping to Chinese Spring compared to when mapping to the pantranscriptome reference (Fig. 5a). This may arise if, when using the Chinese Spring reference, RNA-seq reads were incorrectly assigned to a gene because the correct gene is too divergent and then, when using the pantranscriptome reference, those incorrectly assigned reads now have another more appropriate gene to be assigned to, resulting in fewer reads assigned to the first gene.

While this shows that using Chinese Spring as the reference leads to underestimation of many of these genes, it is important to look at the impact of this on the calculated correlation between homoeologues that led to them being classified as genes of interest by He et al. [11]. We found that the SCC score between homoeologues from this set was − 0.0990 when using the Chinese Spring reference and 0.407 using the pantranscriptome reference (Fig. 5b; *p*-value < 2.2e − 16; 95% confidence interval ranges from − 0.603 to − 0.410). Even though this SCC value remains lower than the mean SCC (~ 0.8) reported for the entire set of homoeologues [11], it indicates that the usage of pantranscriptome as reference increases expression correlation estimates between homoeologues compared to single reference estimates.

**Fig. 6:**
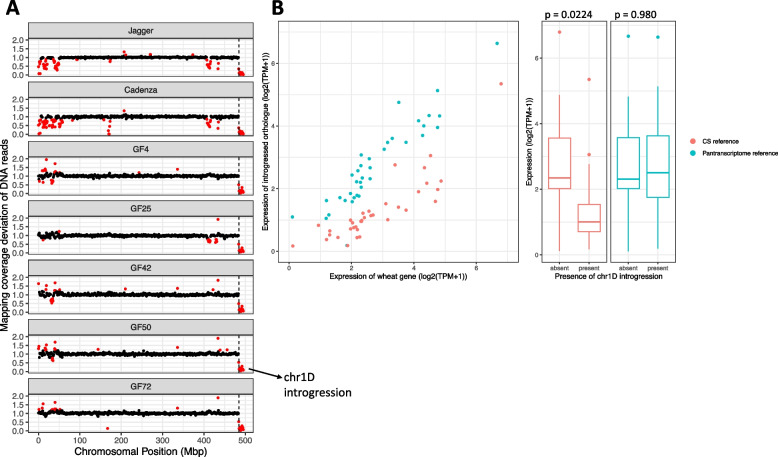
Introgressed genes falsely identified as being less expressed due to reference bias. **A** Mapping coverage deviation of DNA reads across chr1D of Jagger, Cadenza, and 5 of the accessions analysed by He et al. [11]. Each point is the coverage deviation value for a given 1-Mbp genomic window. Windows with a normalised coverage score significantly different to the median normalised coverage score for that window across the set of lines being compared are coloured red. Coverage deviation values significantly below one indicates divergent material is present or a deletion has taken place, relative to the median of the rest of the set of lines. Coverage deviation values and significance values were calculated separately for the accessions and for the cultivars Jagger and Cadenza, the latter two being compared to mapping coverage values from the other cultivars whose genomes were assembled as part of the 10+ wheat genomes project [20]. The reduced coverage at the end of chr1D, the left-hand border of which is indicated by the vertical dashed black line, is the chr1D introgression, common to 53 of the 198 accessions and Jagger and Cadenza which were assembled as part of the 10+ wheat genomes project. **B** Expression of the wheat gene compared to its introgressed orthologue from the chr1D introgression, using either Chinese Spring or the pantranscriptome reference as targets for kallisto pseudoalignment. Orthologue pairs with TPM < 1 in both the introgressed and the wheat copy when mapping to the pantranscriptome reference were excluded. The significance of the difference between introgressed and non-introgressed orthologues when using the Chinese Spring or the pantranscriptome reference was calculated using two-tailed *t* tests with no assumption of equal variance

Several regions with poor mapping coverage (mapping coverage deviation significantly below 1) in multiple accessions overlap precisely with previously identified introgressions from cultivars assembled in the 10+ wheat genomes project [20]. One such introgression is at the end of chr1D (484,302,410–495,453,186 bp, based on RefSeq v1.0 coordinates), present unbroken in 53/198 (26.8%) accessions (Additional file 1: Table S3) and shared with cultivars Jagger and Cadenza (Fig. 6a). The precise overlap of the blocks of the reduced mapping coverage in the accessions and in Jagger and Cadenza suggests that this introgression has the same origin in all these lines, and that no recombination has taken place within the introgression since its introduction. This lack of variation in its size makes it a good candidate for the following analysis. Additionally, this region was highlighted by He et al. [11] as it contains 6 of the genes showing lack of expression correlation, including *ELF3-D1*, which was used as a case example due to its role in heading date [27]. He et al. [11] suggest this is a terminal deletion; however, Wittern et al. [28] identified that the terminal region, including *ELF3-D1*, is an introgression in Cadenza and Jagger, deriving from either *Triticum timopheevii* or *Aegilops speltoides*, based on the *ELF3-D1* gene model possessing an intronic deletion shared with both of these species. We can exclude *Ae. speltoides* as the donor species as protein alignments between the Jagger introgression and *Ae. speltoides* proteins showed a median protein identity of just 91.6%. As *T. timopheevii* does not have a genome assembly available, we cannot confirm it is the donor; however, the mapping profile of *T. timopheevii* reads to the Jagger genome assembly suggest it is a likely match (Additional file 2: Fig. S3). As we cannot be certain about the donor species, we will hereafter refer to this introgression as the chr1D introgression.

We compared the mean expression of genes from the chr1D introgression across accessions that possess the introgression to their 1-to-1 wheat orthologue across the accessions lacking the introgression. When using the Chinese Spring reference, the introgressed genes appear to be less expressed than their wheat orthologues (*p*-value = 0.0224, 95% confidence interval ranges from − 8.65 to − 0.679); however, when using the pantranscriptome reference, no significant difference in expression was found between the genes (Fig. 6b, Additional file 1: Table S4; *p*-value = 0.980, 95% confidence interval ranges from − 4.94 to 4.82).

**Fig. 7:**
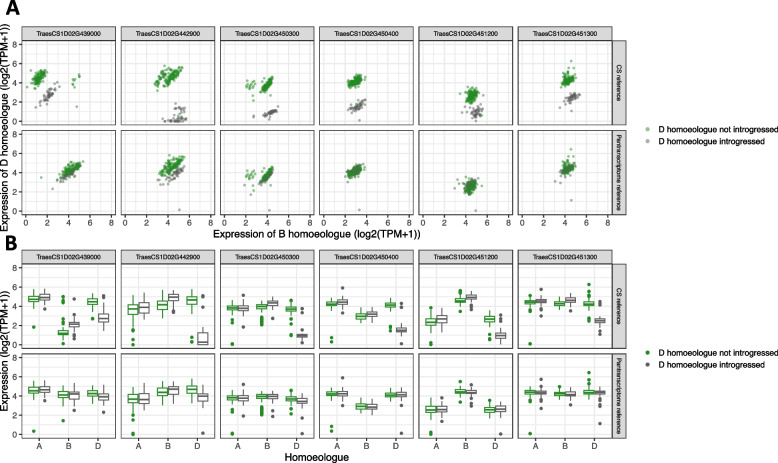
The impact of reference bias on the quantification of triads in which one homoeologue has been introgressed. **A** Estimated expression level of introgressed D homoeologues compared to the wheat B homoeologues and wheat D homoeologues compared to wheat B homoeologues, using either Chinese Spring or pantranscriptome reference as targets for kallisto pseudoalignment. Each point represents one accession. **B** Expression level of triads from where the D homoeologue is an introgressed gene in a subset of lines, using either Chinese Spring or the pantranscriptome reference as targets for kallisto pseudoalignment. The centre line of the boxplots = the median; the box limits = the upper and lower quartiles, the whiskers = 1.5 × interquartile range; and the points = outliers

Earlier, using simulated data, we demonstrated that reference bias can lead to incorrect assignment of expression balance across triads. To examine this phenomenon in real data, we examined the estimated expression across triads within the chr1D introgression that are also in the set of genes showing lack of expression correlation identified by He et al. [11]. When the RNA-seq reads are pseudoaligned to Chinese Spring, in lines with the chr1D introgression, *ELF3-D1* appears to be lowly expressed and the expression of *ELF3-B1* appears slightly elevated compared to accessions without the chr1D introgression. However, when mapped to the pantranscriptome reference, the expression of *ELF3-D1* and *ELF3-B1* in accessions with the chr1D introgression appears very similar to that in accessions without the chr1D introgression (Fig. 7a, b). The CDS sequence for *ELF3-D1* from the introgression shares 97.0% sequence identity with *ELF3-D1* in Chinese Spring, 97.6% identity with *ELF3-A1* and 97.8% identity with *ELF3-B1*. The high divergence of *ELF3-D1* from the introgression and *ELF3-D1* from Chinese Spring and the greater similarity between *ELF3-D1* from the introgression with *ELF3-B1* from Chinese Spring explains how most reads were unable to be assigned, yet some were incorrectly assigned to the *ELF3-B1*, hence the slight increase in estimated expression of *ELF3-B1* when using the Chinese Spring reference. The five other genes showing lack of expression correlation within the chr1D introgression also showed reduced homoeologue imbalance using the pantranscriptome reference and expression level in line with accessions without the chr1D introgression, in which the triad does not contain an introgressed D homoeologue. Four of these genes also showed a slight decrease in estimated expression in the B homoeologue when mapping to the pantranscriptome reference, supporting the idea that false mapping from the introgressed gene to its homoeologue will be driving false negative correlation scores in addition to artificially low expression of the introgressed homoeologue.

## Discussion

In the emerging era of plant pangenomics, chromosome-level assemblies are being generated for an increasing number of cultivars/accessions, which will facilitate a shift away from reference genome-centric methods. Here we have demonstrated the importance of utilising these resources effectively for RNA-seq analyses in wheat to reduce reference bias.

### RNA-seq reference bias in wheat

Quantification of gene expression from RNA-seq reads in wheat is very accurate when the matching reference genome for the sample is available. However, cross-mapping RNA-seq reads leads to detectable levels of reference bias, seen both at the individual gene level and also when assigning triads to categories of homoeologue expression balance. A major cause of this bias appears to be introgressions of diverged gene orthologues from wheat’s wild and domesticated relatives. In some cases, references bias within introgressions could be severe enough to have a strong impact on downstream analyses and conclusion drawn based on these analyses. This analysis was conducted on wheat but other species with substantial introgressed content and/or polyploid genomes may suffer from the same problem. Similar analyses on other species may thus provide value for their respective communities.

Kallisto performed better for self-mapping but when cross-mapping, STAR was better able to deal with divergence between genes, although was far from resolving the issue of reference bias. Similar limitations of alignment-free methods have been previously discussed; for example, Wu et al. [29] demonstrated that kallisto performs poorly for lowly expressed genes and for RNA reads with biological variation compared to the reference.

A future exploration of the impact of reference bias on differential expression calls in wheat will be useful. Reference bias may have little impact on differential expression between conditions or across tissues within a single genotype, as, even if incorrectly quantified, the ratio of estimated expression between conditions/tissues should remain very similar regardless of reference. However, this needs to be assessed formally. If interested in homoeologue expression balance, however, unequal divergence of homoeologues relative to the reference will lead to incorrect findings. Reference bias also makes complex patterns more difficult to discern. For example, in a previous study [30], we demonstrated how the rhythmicity of *ELF3-1D* and *SIG3-1D* in a Cadenza timecourse RNA-seq dataset was difficult to ascertain as the reads mapped so poorly to Chinese Spring. However, when using adding in the introgression to the reference, the reads mapped more correctly, and the rhythmicity could be accurately assessed.

Matching a sample to a more appropriate reference genome will become increasingly possible as genome assemblies for more wheat accessions become available. However, analyses involving two or more accessions require a common reference genome to which the RNA-seq reads can be aligned. In this situation, or when the appropriate genome assembly is not available for within-accession analyses, it is important to exercise caution and check whether introgressed genes might be impacting conclusions drawn. In the long term, it is important to work towards overcoming this issue of introgression-induced reference bias by implementing novel methods.

### Using a pantranscriptome reference to reduce reference bias

Previous work has shown the benefit of using enhanced references or individualised references as targets for RNA-seq mapping. Vijaya Satya, Savaljevski and Reifman [31] constructed an enhanced reference genome for human by including alternative allele segments at known polymorphic loci. Other publications have reported mapping to individualised genomes/transcriptomes by updating the reference with SNPs, INDELs and/or splice sites for each individual [9, 32]. By using individualised genomes instead of a single reference genome, Munger et al. [9] increased the accuracy of eQTL detection in a multi-parent mouse population from 88.2 to 98.3%. Kaminow et al. [33] constructed a pan-human consensus genome by calculating the consensus allele for each variant; this significantly improved the accuracy of RNA-seq mapping when compared to the reference genome. Similar approaches have been used for reducing reference bias when mapping DNA reads [34, 35].

Our approach follows in this vein. However, individualised genomes or consensus genomes are not suitable for wheat as the degree of divergence introduced by introgressions prohibits the accurate genotyping necessary for creating said genomes. Instead, we built a pantranscriptome reference that includes transcripts from other wheat cultivars in the Chinese Spring reference transcriptome. The low resource requirements of kallisto regardless of reference size enables a highly scalable approach as more genome and transcriptome data are generated, while still running in a fraction of the time that alignment-based tools take to align to one reference genome.

The pantranscriptome reference corrects almost all expression values underestimated for genes belonging to an introgression present in the assembled pangenome cultivars and in a 1-to-1 relationship with a Chinese Spring gene. However, this approach does currently have limitations. The pantranscriptome reference will not currently contain all introgressions present across wheat accessions. The pantranscriptome reference is not representative of wheat germplasm around the world; for example, it lacks, with the exception of Chinese Spring, transcripts from Asian and African wheat cultivars. There are several such genomes whose transcripts could be incorporated into the pantranscriptome [36–39]. However, we opted to include only those genomes annotated using the same methodology to ensure accurate orthologue assignment.

As more genomes and/or transcriptomes are sequenced and other existing genomes are re-annotated to provide consistent gene annotations, transcripts can be added to the pantranscriptome reference to broaden the scope of genetic variation covered. This may lead to a saturation point at which most of the commonly segregating variation is captured within the reference and it can be considered complete for most use cases. This approach also only addresses errors caused by divergent genes and not those caused by copy number variation such as tandem duplications, and presence/absence variation caused by a cultivar having a gene deletion or a novel gene. This is because, to ensure additional errors were not introduced, we elected to only add transcripts from other cultivars to the pantranscriptome reference if they came from genes in a 1-to-1 orthologous relationship with a Chinese Spring gene. Developing a way to overcome this limitation is important but also challenging because it requires resolving complex orthologue and paralogue relationships, and it is unclear how novel genes and genes with varying copy number between cultivars should be represented in the pantranscriptome reference.

Different solutions entirely to the problem of RNA-seq reference bias in wheat may emerge as being superior. For example, the field of graph genomes is developing rapidly [40, 41], including methods to align RNA-seq reads to a graph genome [42]. However, graphs for genomes as large and as complex as wheat are yet to be created successfully. It is also a much heavier-weight solution compared to the pantranscriptome pseudoalignment approach. At the very least, our approach provides a temporary way to improve the accuracy of RNA-seq alignment, particularly for those genes comprising the core genome. With further development and the incorporation of new data, it may evolve into an alternative, more lightweight approach to emerging graph-based methods.

### Examining reference bias in experimentally generated RNA-seq data

Using the valuable dataset generated by He et al. [11], we were able to show that reference bias is present in experimentally generated datasets as well as simulated datasets. The diverse nature of the wheat accessions sequenced may have made this work particularly prone to the effects of reference bias; after all, we demonstrated that divergent regions are abundant across the accessions. However, the ubiquity of introgressions is not exclusive to this set of accessions as introgressions are common across most wheat germplasm, including Elite cultivars. Indeed, wheat accessions containing diverse introgressions are very important in wheat research as it may be the source of beneficial variation for breeders, not to mention sources of insight into the evolution of wheat genomes.

The homoeologous sets of genes showing lack of expression correlation identified by He et al. [11] were enriched in genomic regions identified as introgressed or deleted in many of the accessions with 78.2% falling in such regions. We also showed that most of these genes had much higher expression when using the pantranscriptome reference instead of the Chinese Spring reference. Using the pantranscriptome reference also increased the SCC scores calculated between homoeologue pairs. These findings may alter the interpretation of why these genes are associated with productivity traits. While some of these triads may still exhibit genuine dysregulation of homoeologues and homoeologue dosage effects, it is likely that, for at least some of these genes, variation in the gene sequence itself is underlying this trait variation, rather than alteration of expression dosage between homoeologues. This also has implications for the evolutionary and selection mechanisms implicated in the control of these traits.

To more precisely examine how the quantification of introgressed genes changes with the reference used, we focused on genes in the chr1D introgression due to its presence in around a quarter of the accessions and constant size across accessions possessing it. We showed that when using Chinese Spring as the reference, it appears as though introgressed genes are less expressed than the wheat orthologues they replaced. However, when using the pantranscriptome reference, which contains the introgressed gene models as the cultivar Jagger also contains this introgression, there is no significant difference between the expression of these genes. Correcting the quantification of these genes also altered the estimated expression balance across triads in which the D homoeologue is introgressed by raising the estimated expression of the D homoeologue. It would not have been surprising to see, even after removing reference bias, that introgressed genes were expressed differently than the wheat orthologue they replace, perhaps due to the divergence in regulatory sequences. However, this finding suggests that, at least for this introgression, that is not the case. This has implications for any RNA-seq studies using wheat accessions containing introgressions, and also more specifically for studies looking at the expression of introgressed genes and what mechanisms underlie the phenotype they confer.

## Conclusions

Our results highlight the problem of reference bias in wheat RNA-seq alignment which, when relying on a single reference genome, lead to inaccurate gene expression quantification and incorrect assignment of homoeologue expression balance. This effect was shown using both simulated and experimentally generated data. As divergent introgressed genes play a major role in this reference bias, incorporating divergent gene models from different wheat cultivars into the transcriptome reference reduced the extent of reference bias and provides a novel method which can be further developed as high-quality genome assemblies become available for more cultivars.

## Methods

### Read simulation, alignment and quantification

Reads were simulated from the longest transcript from each HC gene in Chinese Spring RefSeq v1.0 [43] (with RefSeq v1.1 annotation) and the nine pseudomolecule genome assemblies [22] if the transcript ≥ 500 bp. Wgsim from samtools v1.9 [44] was used to simulate 1000 pairs of 150 bp reads per gene with an insert size of 400 bp and no errors.

The kallisto index was produced from the CDS sequences from the RefSeq v1.1 high-confidence gene annotations using kallisto v0.44.0 [3]. Reads were pseudoaligned to this index using 100 bootstraps and default settings. Read counts and TPM values were summed across transcripts to generate gene level counts and TPM values.

To construct the pantranscriptome reference, we first ran Orthofinder [25] with standard parameters to define orthogroups based on the longest isoform protein sequences of the HC genes from Chinese Spring and the nine cultivars for which chromosome-level genome assemblies were generated as part of the 10+ genome project [20]. If a gene was found in a 1-to-1 relationship with a Chinese Spring gene, its transcripts were added to the Chinese Spring RefSeq v1.1 HC transcript fasta file. A kallisto index was built and reads pseudoaligned as above. Read counts and TPMs were each summed across all transcripts of a gene and its 1-to-1 orthologues using the custom python script *sum_orthologue_transcript_counts.py* [53] to generate gene-level counts.

The STAR index was built for RefSeq v1.0 with the RefSeq v1.1 HC gene annotation using STAR v2.7.6a [2] using default parameters except for –limitGenomeGenerateRAM 200000000000 and –genomeSAindexNbases 12. The simulated reads from the 10 cultivars were aligned to this index using STAR and the predicted splice junctions from all were merged and then filtered to remove non-canonical junctions, junctions supported by 2 or fewer uniquely mapping reads and reads already annotated in the original genome annotation. The index was rebuilt using these discovered splice sites in addition to the annotated splice sites. The simulated reads from the 10 cultivars were aligned to this new index with parameters –quantMode TranscriptomeSAM and –outSAMunmapped Within. Gene-level read counts were generated using RSEM v1.2.28 [45].

For read count comparisons between self-mapping and cross-mapping, the following criteria were used to determine whether a gene was present in the analysis. For self-mapping, all genes from which reads were simulated were used. For cross-mapping, genes from which reads were simulated in that cultivar and that are in a 1-to-1 relationship with a gene in Chinese Spring from which reads were also simulated were used.

### Defining triad balance

Triads in Chinese Spring were taken from Ramírez-González et al. [10]. For each cultivar, triads were retained if all three homoeologues were used to simulate RNA-seq reads. Triad balance was computed in the same way as [10] except for the use of read counts rather than TPMs due to the way we simulated the reads. The relative read count of each homoeologue within a triad was calculated as follows:

$${A}_{norm}=\frac{A}{A+B+D}$$

$${B}_{norm}=\frac{B}{A+B+D}$$

$${D}_{norm}=\frac{D}{A+B+D}$$

where A, B and D are the read counts of the A, B and D homoeologues, respectively. Euclidean distance was then used to calculate the distance between each set of normalised expression values across a triad to an ideal normalised read count bias for each of seven categories (Table 1). A triad is assigned to an expression bias category by selecting the category with the shortest Euclidean distance between the observed and the ideal bias.

**Table 1:** Ideal normalised read count bias for each triad expression category

Category	A	B	D
Balanced	0.33	0.33	0.33
A suppressed	0	0.5	0.5
B suppressed	0.5	0	0.5
D suppressed	0.5	0.5	0
A dominant	1	0	0
B dominant	0	1	0
D dominant	0	0	1

### Calculating CDS identity

Blastn from blast + v2.7.1 [46] was used to align the nucleotide sequence of the longest transcripts of pairs of orthologues between Chinese Spring RefSeq v1.1 and Lancer. The identity of the best hit between pairs was taken and binned into 5-Mbp genomic windows.

### Binning incorrectly quantified genes

The RefSeq v1.0 genome [43] was split into 5-Mbp genomic windows using bedtools makewindows [47] and for each window, a score was calculated based on the number of under (read count < 500) and overestimated (read count > 1500) genes within that window:

$$\left(-1\ast no.of\;underestimated\;genes\right)+(1\ast no.overestimated\;genes)$$

### Processing sequencing data generated by He et al. [11]

One hundred ninety-eight accessions had both leaf RNA-seq data and enrichment capture short paired-end DNA reads. The RNA-seq data from the 198 lines was pseudoaligned to both Chinese Spring RefSeq v1.1 and the pantranscriptome reference as above for the simulated reads. TPMs were summed across transcripts to generate gene level counts. Accessions GF25, GF270, GF32, GF37, GF41 and GF73 were excluded for RNA-seq analyses as in [11].

DNA reads were mapped to Chinese Spring RefSeq v1.0 [43]. The alignment was filtered using samtools [44]: supplementary alignments, improperly paired reads, and non-uniquely mapped reads (mapping quality less than 10) were removed. PCR duplicates were detected and removed using the Picard Tools v2.1.1 MarkDuplicates function [48]. Accessions GF294, GF342, GF366, GF380, GF381, GF383 and GF38 were excluded for DNA analyses as in [11].

### Using mapping coverage deviation to identify divergent regions of the genome

To generate DNA sequencing reads for the cultivars assembled as part of the 10+ wheat genomes project, we simulated paired-end 150-bp reads with 500-bp insert and no errors from all fourteen *Triticum aestivum* genome assemblies (ArinaLrFor, Cadenza, Claire, Jagger, Julius, Lancer, Landmark, Mace, Norin61, Paragon, Robigus, Stanley, SY Mattis and Weebil) [20] to a depth of 10x using WGSim within samtools v1.9 [44]. Reads were mapped to RefSeq v1.0 as above.

The RefSeq v1.0 genome [43] was split into 1-Mbp genomic windows using bedtools makewindows [47]. Using the filtered read mappings for the cultivars from the 10+ wheat genomes [20] project and for the accessions analysed by He et al. [11], the number of reads mapping to each window was computed using hts-nim-tools [49]. To normalise by the sequencing depth of each line, read counts were divided by the number of mapped reads that passed the filters, producing normalised read counts. Different windows of the genome have variable mapping coverage rates, so to compute coverage deviation we must compare each window to the same window in the other lines in the collection. Median normalised read counts, m, were produced, containing the median for each genomic window. Mapping coverage deviation was then defined for each line as:

$$d_i=\frac{C_i}{m_i\cdot\varepsilon}$$

for window *i* ∈ {1, 2, …, *n*}, where *ε* is the median *d* value across the genome for the line. Statistically significant *d* values were calculated using the scores function from the R package ‘outliers’ using median absolute deviation and probability of 0.99. Mapping coverage deviation and significance values were computed separately for the cultivars from the 10+ wheat genomes project [20] and for the accessions analysed by He et al. [11].

### Locating coordinates of introgression boundaries

To detect the precise locations of the chr1D, chr2A *Ae. ventricosa*, and the chr2D *Ae. markgrafii* introgressions in Jagger, and the chr2B *T. timopheevii* and the chr3D *Th. ponticum* introgression in Lancer, I used the alignments for the simulated Jagger and Lancer reads generated above. Read depths were binned into 5- and 1-Mbp windows using bedtools makewindows [47] and hts-nim-tools [49]. The window in which read depth drops, signifying the start/end of the introgression, was identified for each introgression and IGV was used to precisely identify the position where the coverage profile changes. To locate the location of the introgressions relative to the Jagger/Lancer genomes in order to identify which genes have been introgressed, I extracted Chinese Spring sequence 1Mbp either side of the precisely located border position (or until the end of the chromosome) for each introgression and aligned them to the Jagger or Lancer genome assembly using minimap2 [50] with parameters -x asm5. These alignments were used to determine the borders of the introgressed region as they appear in their donor genomes.

### Characterising the chr1D introgression donor species

Blastp from blast + v2.7.1 [46] was used to align the *Ae. speltoides* proteins with the longest isoforms of the Jagger HC proteins. The best hit for each Jagger protein was kept. Paired-end Illumina DNA reads from *T. timopheevii* [51] were mapped to Chinese Spring RefSeq v1.0 [43] using BWA mem v0.7.13 [52]. Samtools v1.4 [44] was used to filter the alignments to retain mapped reads, primary alignments, properly paired reads and uniquely mapping reads (mapping quality greater than 10). PCR duplicates were found and removed using the Picard Tools v2.1.1 MarkDuplicates function [48]. Read depths were binned into 5-Mbp windows using bedtools makewindows [47] and hts-nim-tools [49] and divided by window length to account for windows at ends of chromosomes which are less than 5Mbp in length.

### Calculating SCC between homoeologues

SCC scores were calculated between AB, AD and BD homoeologue pairs for triads where one homoeologue was in the set of genes showing lack of expression correlation identified by He et al. [11]. This was done using the cor.test function in R with the ‘Spearman’ method and the lowest SCC value of the three comparisons was taken. Triads were excluded if any of the homoeologues were not found in the HC RefSeq v1.1 annotation.

### Statistical tests

The significance of the difference in the proportion of genes that were correctly quantified between introgressed and non-introgressed regions was calculated using a chi-squared test with a sample size of 60,338. The significance of the difference between mean CDS nucleotide identity between orthologue pairs when correctly quantified compared to incorrectly quantified was calculated using two-tailed *t* tests with no assumption of equal variance and a sample size of 60,338. The significance of the difference in Spearman correlation scores between homoeologue pairs when using the Chinese Spring reference compared to the pantranscriptome reference was calculated using a two-tailed *t* test with no assumption of equal variance and a sample of 55. The significance of the difference between introgressed and non-introgressed orthologues when using the Chinese Spring or the pantranscriptome reference was calculated using two-tailed *t* tests with no assumption of equal variance with a sample size of 63.

### Supplementary Information

**Additional file 1: Table S1.** Number of genes correctly quantified, underestimated, and overestimated from simulated RNA-Seq data, using Kallisto with the Chinese Spring reference, STAR with the Chinese Spring reference, or kallisto with the pantranscriptome reference. **Table S2.** Percentage of triads classified in each expression category from simulated RNA-Seq data, using Kallisto with the Chinese Spring reference, STAR with the Chinese Spring reference, or kallisto with the pantranscriptome reference. **Table S3.** Accessions from the He *et al*. [11] dataset that do and do not contain the chr1D introgression. **Table S4.** Expression values for genes within the chr1D introgression for accessions from the He *et al*. [11] dataset, using either the Chinese Spring or the pantranscriptome reference. Accessions are split based on whether or not they contain the chr1D introgression.

**Additional file 2: Fig. S1.** Upset plot of 1-to-1 orthologue assignments used for the construction of the pantranscriptome reference. **Fig. S2.** Remaining incorrectly quantified genes after correction using the pantranscriptome reference. **Fig. S3.** Reads from *T. timopheevii* accession P95 mapped to *T. aestivum* cv. Jagger and binned into 5Mbp genomic windows.

## Data Availability

The pantranscriptome reference, along with a python script to sum expression counts across all transcripts of a given Chinese Spring gene and its 1-to-1 orthologues, can be accessed via figshare at 10.6084/m9.figshare.24242767 [53]. The RNA-seq data and DNA sequencing data generated by He et al. [11] are stored in the European Nucleotide Archive under project codes PRJNA670223 [54] and PRJNA787276 [55]. The wheat cultivar genomes and annotations generated as part of the 10+ wheat genomes project [20] can be accessed on Ensembl Plants release 58 via https://plants.ensembl.org/Triticum_aestivum/Info/Cultivars [56].

## Abbreviations

eQTL: Expression quantitative trait locus
HC: High confidence
SCC: Spearman’s correlation coefficient

## References

[1] Kim D, Paggi JM, Park C, Bennett C, Salzberg SL (2019). Graph-based genome alignment and genotyping with HISAT2 and HISAT-genotype. Nat Biotechnol.

[2] Dobin A, Davis CA, Schlesinger F, Drenkow J, Zaleski C, Jha S (2013). STAR: Ultrafast universal RNA-seq aligner. Bioinformatics.

[3] Bray NL, Pimentel H, Melsted P, Pachter L (2016). Near-optimal probabilistic RNA-seq quantification. Nat Biotechnol.

[4] Patro R, Duggal G, Love MI, Irizarry RA, Kingsford C (2017). Salmon provides fast and bias-aware quantification of transcript expression. Nat Methods.

[5] Günther T, Nettelblad C (2019). The presence and impact of reference bias on population genomic studies of prehistoric human populations. PLoS Genet.

[6] Thorburn DMJ, Sagonas K, Binzer-Panchal M, Chain FJJ, Feulner PGD, Bornberg-Bauer E (2023). Origin matters: Using a local reference genome improves measures in population genomics. Mol Ecol Resour..

[7] Zhan S, Griswold C, Lukens L (2021). Zea mays RNA-seq estimated transcript abundances are strongly affected by read mapping bias. BMC Genomics.

[8] Li L, Petsch K, Shimizu R, Liu S, Xu WW, Ying K (2013). Mendelian and non-mendelian regulation of gene expression in Maize. PLoS Genet.

[9] Munger SC, Raghupathy N, Choi K, Simons AK, Gatti DM, Hinerfeld DA (2014). RNA-Seq alignment to individualized genomes improves transcript abundance estimates in multiparent populations. Genetics.

[10] Ramírez-González RH, Borrill P, Lang D, Harrington SA, Brinton J, Venturini L, et al*.* The transcriptional landscape of polyploid wheat. Science. 2018; 361(6403):eaar6089. 10.1126/science.aar6089 30115782

[11] He F, Wang W, Rutter WB, Jordan KW, Ren J, Taagen E, DeWitt N, Sehgal D, Sukumaran S, Dreisigacker S, Reynolds M, Halder J, Sehgal SK, Liu S, Chen J, Fritz A, Cook J, Brown-Guedira G, Pumphrey M, Carter A, Sorrells M, Dubcovsky J, Hayden MJ, Akhunova A, Morrell PL, Szabo L, Rouse M, Akhunov E. Genomic variants affecting homoeologous gene expression dosage contribute to agronomic trait variation in allopolyploid wheat. Nat Commun. 2022;13(826). 10.1038/s41467-022-28453-y. 10.1038/s41467-022-28453-y PMC8837796 35149708

[12] Edelman NB, Mallet J (2021). Prevalence and adaptive impact of introgression. Ann Rev Genet.

[13] Mallet J (2005). Hybridization as an invasion of the genome. Trends Ecol Evol.

[14] Hao M, Zhang L, Ning S, Huang L, Yuan Z, Wu B (2020). The resurgence of introgression breeding, as exemplified in wheat improvement. Front Plant Sci.

[15] Zhou Y, Zhao X, Li Y, Xu J, Bi A, Kang L (2020). Triticum population sequencing provides insights into wheat adaptation. Nat Genet.

[16] Cheng J, Liu J, Wen J, Nie X, Xu L, Chen N, Li Z, Wang Q, Zheng Z, Li M, Cui L, Liu Z, Bian J, Wang Z, Xu S, Yang Q, Appels R, Han D, Song W, Sun Q, Jiang Y. Frequency intra- and inter-species introgression shapes the landscape of genetic variation in bread wheat. Genome Biol. 2019;20(136). 10.1186/s13059-019-1744-x. 10.1186/s13059-019-1744-x PMC6624984 31300020

[17] He F, Pasam R, Shi F, Kant S, Keeble-Gagnere G, Kay P (2019). Exome sequencing highlights the role of wild-relative introgression in shaping the adaptive landscape of the wheat genome. Nat Genet.

[18] Przewieslik-Allen AM, Burridge AJ, Wilkinson PA, Winfield MO, Shaw DS, McAusland L (2019). Developing a High-Throughput SNP-based marker system to facilitate the introgression of traits from aegilops species into bread wheat (Triticum aestivum). Front Plant Sci.

[19] Wang Z, Wang W, Xie X, Wang Y, Yang Z, Peng H (2022). Dispersed emergence and protracted domestication of polyploid wheat uncovered by mosaic ancestral haploblock inference. Nat Commun.

[20] Walkowiak S, Gao L, Monat C, Haberer G, Kassa MT, Brinton J (2020). Multiple wheat genomes reveal global variation in modern breeding. Nature.

[21] Keilwagen J, Lehnert H, Berner T, Badaeva E, Himmelbach A, Börner A (2022). Detecting major introgressions in wheat and their putative origins using coverage analysis. Sci Rep.

[22] White B, Lux T, Rusholme-Pilcher R, Kaithakottil G, Duncan S, Simmonds J (2024). De novo annotation of the wheat pan-genome reveals complexity and diversity within the hexaploid wheat pan-transcriptome. BioRxiv.

[23] Dvorak J, Akhunov ED, Akhunov AR, Deal KR, Luo M-C (2006). Molecular characterization of a diagnostic DNA marker for domesticated tetraploid wheat provides evidence for gene flow from wild tetraploid wheat to hexaploid wheat. Mol Biol Evol.

[24] Gao L, Koo D-H, Juliana P, Rife T, Singh D, Lemes da Silva C, et al. The Aegilops ventricosa 2NvS segment in bread wheat: cytology, genomics and breeding. Theor Appl Genet. 2021;134(2):529–42. 10.1007/s00122-020-03712-y PMC7843486 33184704

[25] Emms DM, Kelly S (2019). OrthoFinder: phylogenetic orthology inference for comparative genomics. Genome Biol.

[26] Srivastava A, Malik L, Sarkar H, Zakeri M, Almodaresi F, Soneson C (2020). Alignment and mapping methodology influence transcript abundance estimation. Genome Biol.

[27] Wang J, Wen W, Hanif M, Xia X, Wang H, Liu S (2016). TaELF3-1DL, a homolog of ELF3, is associated with heading date in bread wheat. Mol Breed.

[28] Wittern L, Steed G, Taylor LJ, Ramirez DC, Pingarron-Cardenas G, Gardner K (2023). Wheat EARLY FLOWERING 3 affects heading date without disrupting circadian oscillations. Plant Physiol.

[29] Wu DC, Yao J, Ho KS, Lambowitz AM, Wilke CO (2018). Limitations of alignment-free tools in total RNA-seq quantification. BMC Genomics.

[30] Rees H, Rusholme-Pilcher R, Bailey P, Colmer J, White B, Reynolds C (2022). Circadian regulation of the transcriptome in a complex polyploid crop. PLoS Biol.

[31] Vijaya Satya R, Zavaljevski N, Reifman J (2012). A new strategy to reduce allelic bias in RNA-Seq readmapping. Nucleic Acids Res.

[32] Liu X, MacLeod JN, Liu J (2018). iMapSplice: Alleviating reference bias through personalized RNA-seq alignment. PLoS ONE.

[33] Kaminow B, Ballouz S, Gillis J, Dobin A (2022). Pan-human consensus genome significantly improves the accuracy of RNA-seq analyses. Genome Res.

[34] Chen NC, Solomon B, Mun T, Iyer S, Langmead B (2021). Reference flow: reducing reference bias using multiple population genomes. Genome Biol.

[35] Vaddadi NSK, Mun T, Langmead B. Minimizing Reference Bias with an Impute-First Approach. bioRxiv. 2023. 10.1101/2023.

[36] Athiyannan N, Abrouk M, Boshoff WHP, Cauet S, Rodde N, Kudrna D (2022). Long-read genome sequencing of bread wheat facilitates disease resistance gene cloning. Nat Genet.

[37] Guo W, Xin M, Wang Z, Yao Y, Hu Z, Song W (2020). Origin and adaptation to high altitude of Tibetan semi-wild wheat. Nat Commun.

[38] Shi X, Cui F, Han X, He Y, Zhao L, Zhang N (2022). Comparative genomic and transcriptomic analyses uncover the molecular basis of high nitrogen-use efficiency in the wheat cultivar Kenong 9204. Mol Plant.

[39] Jia J, Zhao G, Li D, Wang K, Kong C, Deng P (2023). Genome resources for the elite bread wheat cultivar Aikang 58 and mining of elite homeologous haplotypes for accelerating wheat improvement. Mol Plant.

[40] Garrison E, Sirén J, Novak AM, Hickey G, Eizenga JM, Dawson ET (2018). Variation graph toolkit improves read mapping by representing genetic variation in the reference. Nat Biotechnol.

[41] Martiniano R, Garrison E, Jones ER, Manica A, Durbin R (2020). Removing reference bias and improving indel calling in ancient DNA data analysis by mapping to a sequence variation graph. Genome Biol.

[42] Sibbesen JA, Eizenga JM, Novak AM, Sirén J, Chang X, Garrison E (2023). Haplotype-aware pantranscriptome analyses using spliced pangenome graphs. Nat Methods.

[43] Appels R, Eversole K, Feuillet C, Keller B, Rogers J, Stein N, et al. Shifting the limits in wheat research and breeding using a fully annotated reference genome. Science. 2018;361(6403):eaar7191. 10.1126/science.aar7191 30115783

[44] Li H, Handsaker B, Wysoker A, Fennell T, Ruan J, Homer N (2009). The Sequence Alignment/Map format and SAMtools. Bioinformatics.

[45] Li B, Dewey CN (2011). RSEM: Accurate transcript quantification from RNA-Seq data with or without a reference genome. BMC Bioinformatics.

[46] Camacho C, Coulouris G, Avagyan V, Ma N, Papadopoulos J, Bealer K (2009). BLAST+: Architecture and applications. BMC Bioinformatics.

[47] Quinlan AR, Hall IM (2010). BEDTools: a flexible suite of utilities for comparing genomic features. Bioinformatics.

[48] Depristo MA, Banks E, Poplin R, Garimella KV, Maguire JR, Hartl C (2011). A framework for variation discovery and genotyping using next-generation DNA sequencing data. Nat Genet.

[49] Pedersen BS, Quinlan AR (2018). hts-nim: scripting high-performance genomic analyses. Bioinformatics.

[50] Li H (2018). Minimap2: pairwise alignment for nucleotide sequences. Bioinformatics.

[51] King J, Grewal S, Othmeni M, Coombes B, Yang CY, Walter N, Ashling S, Scholefield D, Walker J, Hubbart-Edwards S, Hall A, King IP. Introgression of the Triticum timopheevii Genome Into Wheat Detected by Chromosome-Specific Kompetitive Allele Specific PCR Markers. Front Plant Sci. 2022;13(919519). 10.3389/fpls.2022.919519. 10.3389/fpls.2022.919519 PMC9198554 35720607

[52] Li H, Durbin R (2009). Fast and accurate short read alignment with Burrows-Wheeler transform. Bioinformatics.

[53] Coombes B, Lux T, Akhunov E, Hall A. Supplementary Data for paper titled 'Introgressions lead to reference bias in wheat RNA-Seq analysis'. 2023. figshare 10.6084/m9.figshare.24242767.v1. 10.1186/s12915-024-01853-w PMC10921782 38454464

[54] RNA-seq data for a wheat diversity panel. ENA https://www.ebi.ac.uk/ena/browser/view/PRJNA670223 (2022).

[55] Regulatory sequence diversity in the wheat genome. ENA https://www.ebi.ac.uk/ena/browser/view/PRJNA787276 (2020).

[56] Yates DY, Allen J, Amode RM, Azov AG, Barba M, Becerra A (2022). Ensembl Genomes 2022: an expanding genome resource for non-vertebrates. Nucleic Acids Res.

