# Supplementary material for: Introgressions lead to reference bias in wheat RNA-seq analysis
Source: BMC Biol. 2024 Mar 7;22:56. doi: 10.1186/s12915-024-01853-w (PMC10921782; doi:10.1186/s12915-024-01853-w)
Supplement: Supplementary file 2 — Additional file 2: Fig. S1. Upset plot of 1-to-1 orthologue assignments used for the construction of the pantranscriptome reference. Fig. S2. Remaining incorrectly quantified genes after correction using the pantranscriptome reference. Fig. S3. Reads from T. timopheevii accession P95 mapped to T. aestivum cv. Jagger and binned into 5Mbp genomic windows. [file 12915_2024_1853_MOESM2_ESM.pdf]

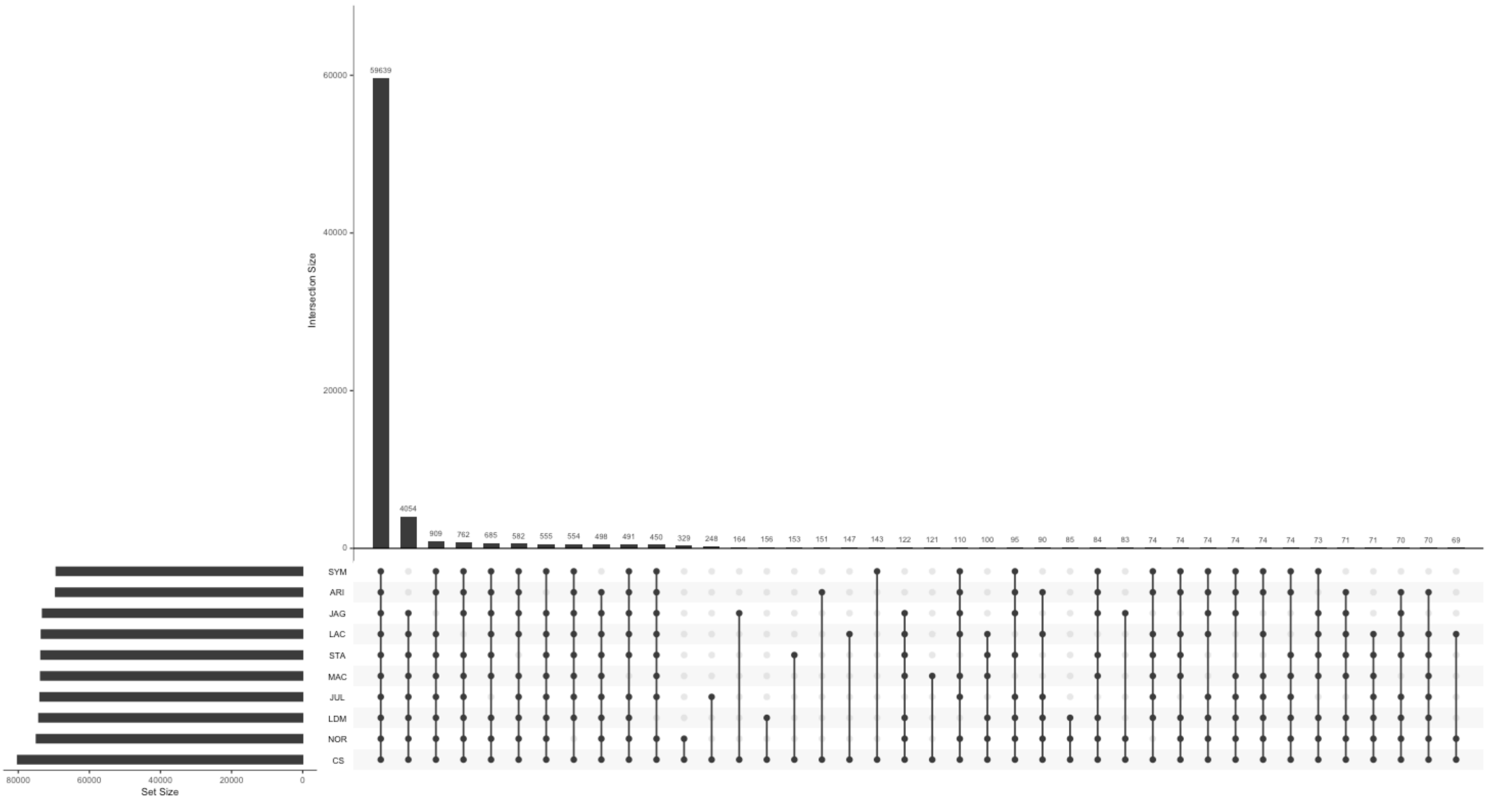


**Figure S1. Upset plot of 1-to-1 orthologue assignments used for the construction of the pantranscriptome reference.**

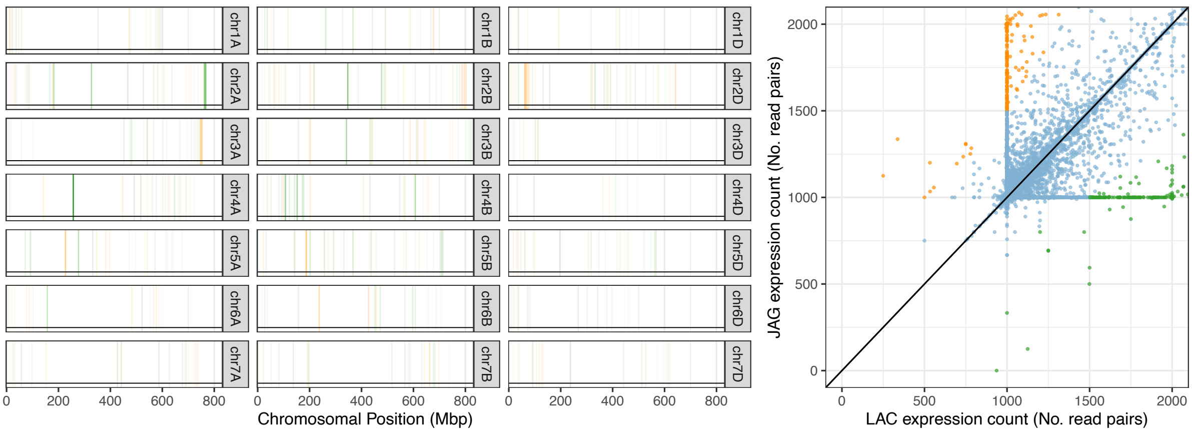


**Figure S2. Remaining incorrectly quantified genes after correction using the pantranscriptome reference.** Scatter plot shows expression counts for simulated reads of Lancer-Jagger orthologue pairs when using kallisto with the pantranscriptome reference. Genes are considered incorrectly quantified if their estimated read count is 1.5x or 1/1.5x the other cultivar. The chromosome plot shows the distribution of incorrectly quantified genes in 5Mbp windows, coloured by the cultivar in which the estimated expression is lower; orange blocks are underestimated in Lancer compared to Jagger, while green blocks are underestimated in Jagger compared to Lancer.

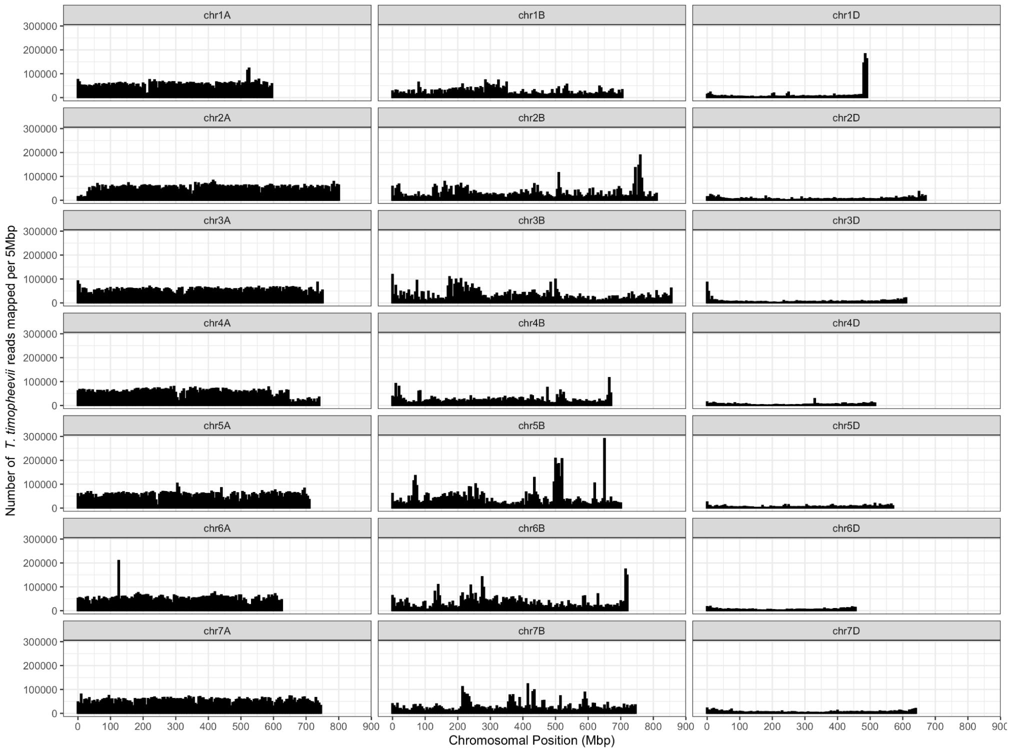


**Figure S3. Reads from *T. timopheevii* accession P95 mapped to *T. aestivum* cv. Jagger and binned into 5Mbp genomic windows.** Number of mapped reads were divided by the length of window to accurately reflect read density at the final window of each chromosome. The chr1D introgression with a putative origin of *T. timopheevii* is at 481585620-493450010. *T. timopheevii* is a tetraploid with genomes related to the A and B subgenome of wheat. This is reflected in the greater mappability of *T. timopheevii* reads to the A and B subgenomes than the D subgenome. However, the higher read count across the 1D introgression suggests this region is more similar between *T. timopheevii* and the introgression than between its subgenomes and the A and B subgenomes, lending support to the donor of this introgression being *T. timopheevii*.
